# Insurance against Surgical Operations

**Published:** 1904-04-30

**Authors:** 


### Editor's Letter-Box.

**[Our Correspondents are reminded that prolixity is a great bar to publication, and that brevity of style and conciseness of statement greatly facilitate early insertion.]**

### INSURANCE AGAINST SURGICAL OPERATIONS.

We have had a large number of inquiries as to the name of the Insurance Company whose prospectus is referred to in the leading article of our issue of March 19th last. The company is the Commercial Union Assurance Company, Limited, 24, 25, and 26 Cornhill, London, E.C.

